# Hepatic Amyloidosis as a Rare Cause of Liver Failure: A Case Report

**DOI:** 10.7759/cureus.27274

**Published:** 2022-07-26

**Authors:** Haider Ghazanfar, Misbahuddin Khaja, Asim Haider, Laura Yapor, Sameer Kandhi, Muhammad Sulh

**Affiliations:** 1 Gastroenterology, BronxCare Health System, Icahn School of Medicine at Mount Sinai, Bronx, USA; 2 Internal Medicine/Pulmonary and Critical Care Medicine, BronxCare Health System, Icahn School of Medicine at Mount Sinai, Bronx, USA; 3 Internal Medicine, BronxCare Health System, Icahn School of Medicine at Mount Sinai, Bronx, USA; 4 Pulmonary and Critical Care Medicine, BronxCare Health System, Icahn School of Medicine at Mount Sinai, Bronx, USA; 5 Internal Medicine, BronxCare Health System, Bronx, USA; 6 Pathology, BronxCare Health System, Icahn School of Medicine at Mount Sinai, Bronx, USA

**Keywords:** renal failure, liver failure, morbidity and mortality, renal amyloidosis, hepatic amyloidosis

## Abstract

Amyloidosis is a systemic disease that results from the extracellular deposition of an abnormal protein called amyloid. The kidney and the heart are the most common organ affected by amyloidosis while in some cases liver involvement can be seen. Our patient is a 60-year-old African American male who presented to the emergency department because of multiple episodes of syncope over the past day. Chest x-ray and ultrasound chest were suggestive of pleural effusion for which thoracentesis was done. His hospital course was complicated with renal and liver failure. Computed tomography (CT) abdomen and pelvis was done which showed mild hepatomegaly. Liver biopsy was done which showed congo red stain positive for amyloid. The patient's clinical condition continued to worsen and he was started on hemodialysis. During hospital course, the patient developed liver failure. His family members opted for palliative care and the patient passed away during the same admission. Physicians need to be aware of the detrimental course and poor prognosis associated with hepatic and renal amyloidosis. High clinical suspicion is needed to make an early diagnosis and initiate prompt treatment. Although clinical, laboratory and radiological findings can help in suggesting amyloidosis, a tissue biopsy is needed to confirm the diagnosis of amyloidosis.

## Introduction

Amyloidosis is a systemic disease that results from the extracellular deposition of an abnormal protein called amyloid. The prevalence of amyloid light chain (AL) amyloidosis has significantly increased by 12% in the United States between 2007 and 2015 [1]. The prevalence of amyloidosis significantly increased with age [2]. Currently, there are 22 different types of localized forms of amyloidosis and 18 different types of systemic forms of amyloidosis. The main subtype of systemic amyloidosis is primary AL amyloidosis, secondary amyloid A (AA) amyloidosis, B2 macroglobulin-related amyloidosis, and familial amyloidosis [3]. Systemic AL amyloidosis is more prevalent in developed countries, while secondary amyloidosis is more prevalent in developing countries. We present a case of a 60-year-old male patient who presented with syncope and was later diagnosed with liver amyloidosis.

## Case presentation

Our patient is a 60-year-old African American male who presented to the emergency department because of multiple episodes of syncope over the past day. He stated that the preceding day, he passed out and fell on the right side of the head. He had two more episodes of syncope, which prompted him to come to the emergency department. He complained of pins and needle sensation on upper extremities and generalized fatigue for the last one week. He also complained of shortness of breath for the past day, which has progressively worsened with time. His past medical history was significant for renal amyloidosis, gastric ulcer, and hyperlipidemia. He was diagnosed with AL renal amyloidosis three months ago. The diagnosis was made on the basis of renal biopsy. The renal biopsy had shown renal amyloidosis, AL-kappa type involving glomeruli, tubulointerstitial, and arteriolar wall. His family history was significant for renal disease in the father. He did not follow-up with his appointments after the diagnosis. He denied smoking or using illicit substances.

In the emergency department, he was found to have blood pressure of 101/63 mm Hg, heart rate of 86 beats per minute, temperature of 38.7°C, and respiratory rate of 18 breaths per minute. He was saturating 92% on room air and a nasal cannula was placed. General physical examination was significant for right periorbital ecchymosis with upper eyelid abrasion and bilateral lower extremity edema. He had decreased breath sounds on the left side. Cardiac examination revealed normal S1 and S2 heart sounds. His abdomen was non-tender on examination. He had normal bowel sound and was found to have hepatomegaly on palpation. He was oriented to time, place, and person and had no focal sensory or motor deficits. His initial laboratory findings have been presented in Table 1.

**Table 1:** Initial laboratory findings of the patient

Investigation	Result
White blood cell count	9.5 (4.8-10.8 k/uL)
Red blood cell count	4.97 (4.50-5.90 MIL/uL)
Hemoglobin	15.6 (12.0-16.0 g/dL)
Hematocrit	47.2 (42-51%)
Platelet	323 (150-400 k/uL)
Sodium, serum	130 (135-145 mEq/L)
Potassium, serum	4.6(3.5-5.0 mEq/L)
Blood urea nitrogen, serum	26 (8-26 mg/dL)
Creatinine, serum	1.6 (0.5-1.5 mg/dL)
Hepatic function panel	
Albumin, serum	1.2 (3.2-4.6 g/dl)
Bilirubin, serum total	1.1 (0.2-1.1 mg/dL)
Bilirubin, serum direct conjugated	0.5 (0.0-0.3 mg/dL)
Alkaline phosphatase, serum	1000 (56-155 unit/L)
Aspartate transaminase, serum	129 (9-48 unit/L)
Alanine aminotransferase, serum	90 (5-40 unit/L)
Lactate dehydrogenase, serum	389 (110-210 unit/L)
Total protein, serum	8.4 (5.8 - 8.3 g/dl)

**Figure 1:**
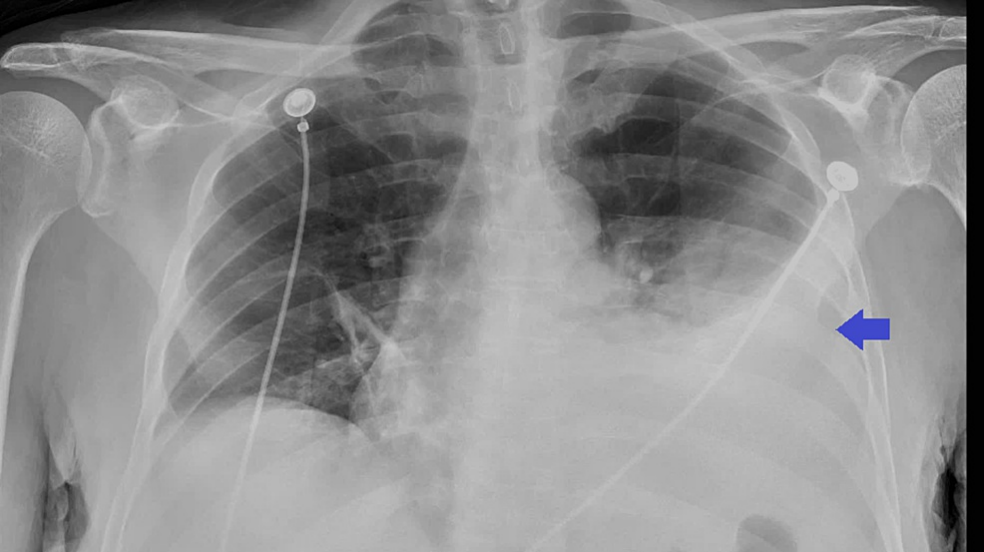
Chest x-ray showing left lung base opacity likely representing a combination of effusion and atelectasis (arrow)

**Figure 2:**
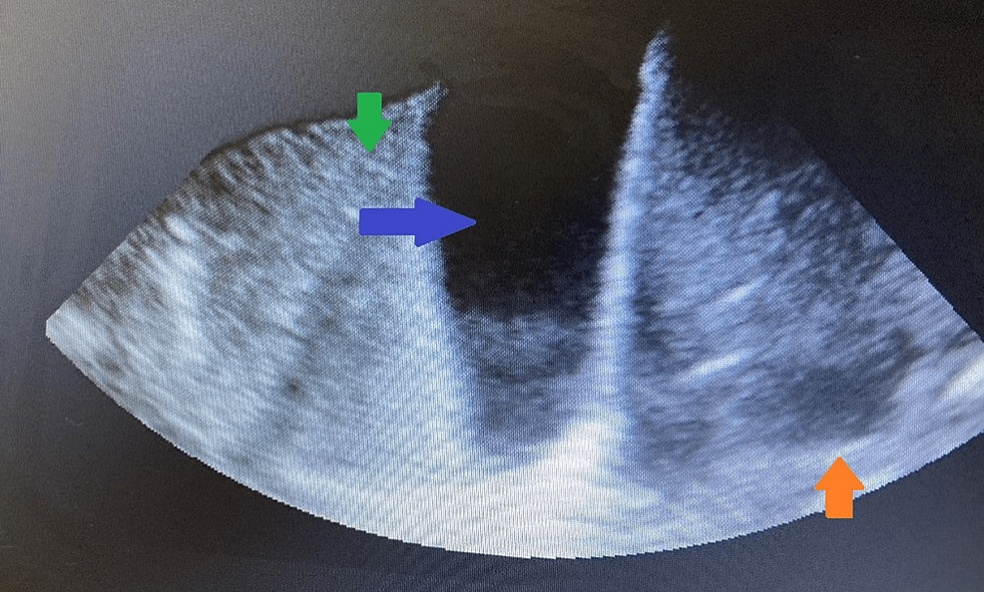
Chest ultrasound showing pleural effusion (blue arrow), liver (orange arrow), and lung (green arrow)

Chest x-ray was remarkable for left lung base opacity likely representing a combination of effusion and atelectasis (Figure 1). He was intubated because of worsening hypoxia and hypercapnia. Chest ultrasound showed pleural effusion (Figure 2).

**Figure 3:**
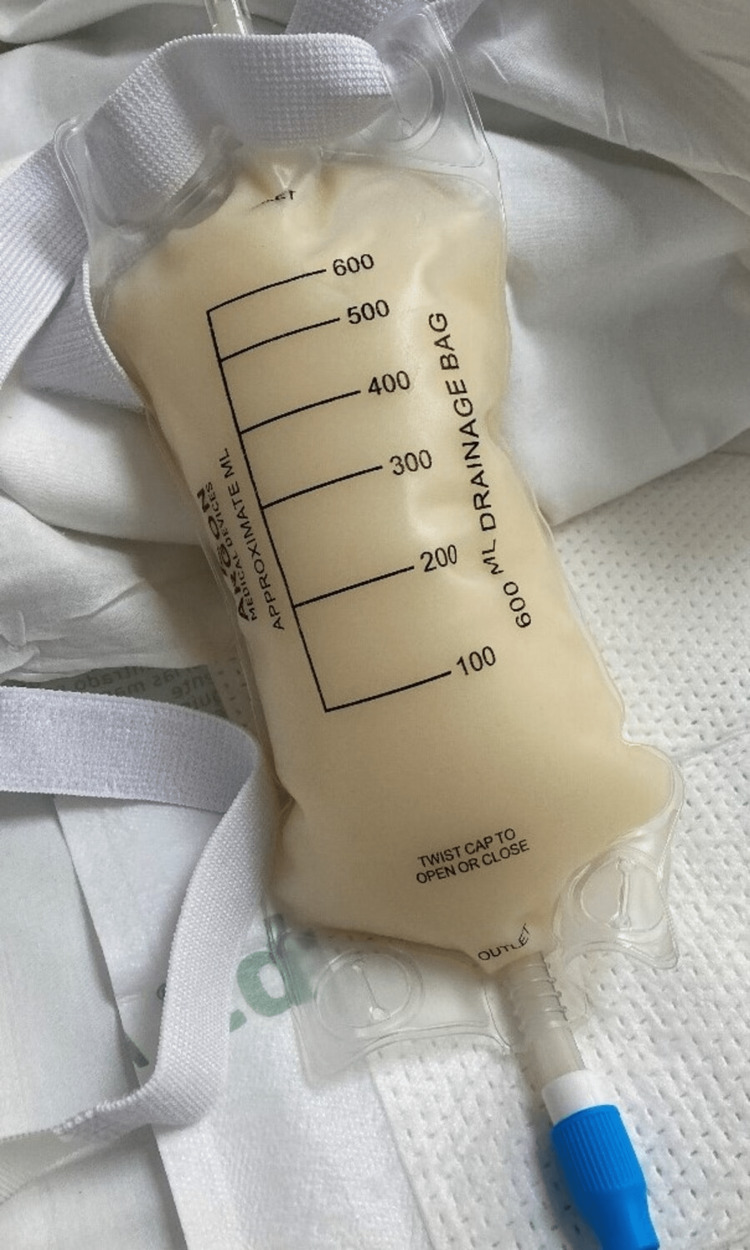
Chylous pleural effusion seen in the drainage bag

Broad spectrum antibiotics were started and he underwent thoracentesis and 1100 cc of milky pleural fluid was drained (Figure 3). Pleural fluid analysis showed transudative effusion and has been presented in Table 2.

**Table 2:** Pleural fluid analysis of the patient

Investigation	Result
Pleural pH	8
Color of pleural fluid	Yellow
Body fluid appearance	Hazy
White blood cell count, pleural fluid (cells/mm^3^)	33
Red blood cell count pleural fluid (mil cells/mm^3^)	11
LDH pleural fluid (unit/L)	43
Triglyceride fluid (mL)	174
Protein pleural fluid (g/dL)	0.3
Albumin pleural fluid (mg/dL)	0.1
Glucose pleural fluid (mg/dL)	154
Amylase pleural fluid (unit/L)	185

His echocardiogram revealed ejection fraction of 78.1%, small left ventricle (LV) cavity size, normal LV diastolic filling, normal left atrium size, normal right ventricle size and function, and large left-sided pleural effusion. He underwent computed tomography (CT) of the head, which showed acute on chronic left subdural hematoma over frontoparietal area and minimally displaced fracture of the right zygomatic arch. He successfully underwent left-sided craniotomy for evacuation of subdural hematoma. His hospital course was complicated with worsening renal function and transaminitis. CT abdomen and pelvis without contrast was done which showed free fluid in the abdomen and pelvis and mild hepatomegaly. 

**Figure 4:**
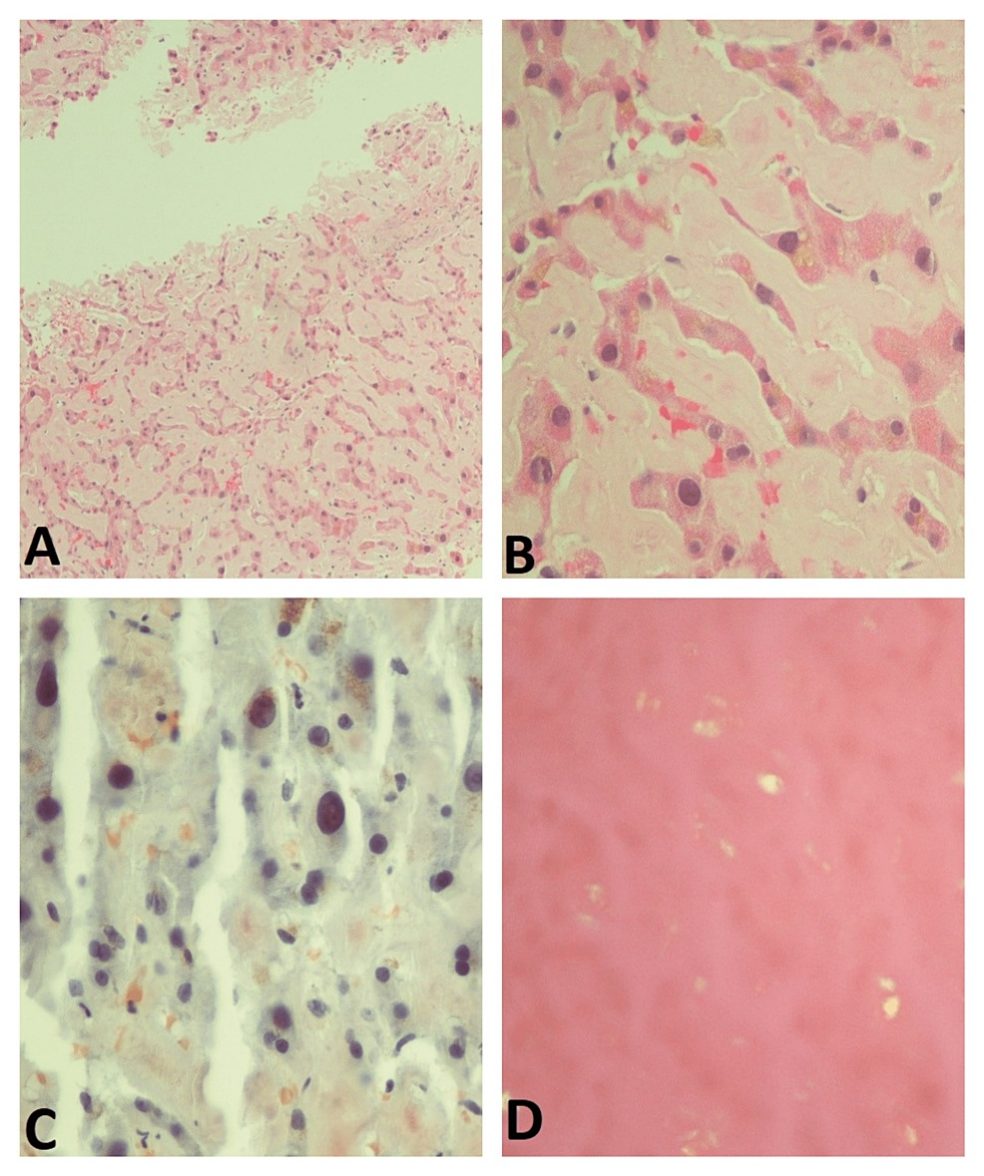
Liver biopsy of the patient (A) Low power and (B) high power showing H&E stain of the liver biopsy shows amorphous, acellular deposits involving perisinusoidal and portal areas with liver cell plates atrophy consistent with amyloidosis. (C) Congo red stain and (D) apple-green birefringence were seen under polarized light.

Liver biopsy was done which showed Congo red stain positive for amyloid (Figures 4A-4D). He was diagnosed with liver amyloidosis. His case was discussed with the liver transplant center, but the patient was found not to be a candidate for liver transplant. Due to worsening renal function, he was started on hemodialysis. The patient's clinical condition continue to worsen and he developed liver failure. Family members opted for palliative care and he underwent palliative extubation later on and passed away on the same day.

## Discussion

Clinical signs and symptoms of amyloidosis depend on the type of precursor protein, tissue distribution, and the amount of amyloid. Gastrointestinal manifestations of amyloidosis include bleeding, gastroparesis, diarrhea, constipation, bacterial overgrowth, malabsorption, hepatomegaly, splenomegaly, and chronic gastrointestinal dysmotility. Kidneys and the heart are the most common organ affected by amyloidosis while liver involvement is seen in only 9% cases of amyloidosis [4]. In patients with hepatic amyloidosis, hepatomegaly is only seen in 57-83% of the cases [5]. Hepatic involvement is more in patients with AL amyloid as compared to AA amyloidosis. Patient with hepatic amyloidosis usually presents with hepatomegaly and elevated alkaline phosphatase level [6]. Our patient had hepatomegaly and highly elevated alkaline phosphatase. A study done on 98 patients found that 86% of patients had elevated serum alkaline phosphatase levels [7]. Hepatic involvement in AL amyloid is due to abnormal excessive deposition of monoclonal immunoglobulin light chains in the parenchymal tissue of the liver. Most of the deposition occurs in the hepatic perisinusoidal space of Disse and within the blood vessel walls [8]. This excessive deposition of amyloid material leads to the atrophy of the hepatocyte which in turn into liver dysfunction and liver failure.

Radiologic findings of hepatic amyloidosis are nonspecific and liver biopsy is needed to confirm the diagnosis. Ultrasound findings in patients with hepatic amyloidosis include hepatomegaly and heterogenous hepatic echotexture [9]. Abdominal CT scans in these patients can show diffuse or focal areas of decreased parenchymal attenuation and prominent calcification in some patients [9]. Our patient had mild hepatomegaly on abdominal CT. MRI of liver shows a high signal intensity on T1 throughout the liver without any significant change on T2 [9]. Some studies have shown that patients with hepatic amyloidosis have a high score of liver stiffness on fibroscan; however, further studies are needed to ascertain the diagnostic value of these findings [10]. Histology showing the Congo red staining of the amyloid fibrils is the gold standard for diagnosis [10].

Patients with systemic amyloidosis have been shown to have survival of 14 months [11]. A study on 98 patients with hepatic amyloidosis showed that the median survival in these patients was 8.5 months [7]. Most of these patients die of decompensated liver cirrhosis, hepatic failure, and secondary infection. Our patient had both renal and hepatic amyloidosis, and he developed both renal and liver failure. Furthermore, pleural effusions in cases of systemic amyloidosis in patients with AL amyloidosis are associated with poor prognosis [12].

## Conclusions

Physicians need to be aware of the detrimental course and poor prognosis associated with hepatic and renal amyloidosis. High clinical suspicion is needed to make an early diagnosis and initiate prompt treatment. Although clinical, laboratory, and radiological findings can help in suggesting amyloidosis, a tissue biopsy is needed to confirm the diagnosis of amyloidosis.
